# rTMS reduces delta and increases theta oscillations in Alzheimer's disease: A visual‐evoked and event‐related potentials study

**DOI:** 10.1111/cns.14564

**Published:** 2024-01-12

**Authors:** Halil Aziz Velioglu, Esra Zeynep Dudukcu, Lutfu Hanoglu, Bahar Guntekin, Tuba Akturk, Burak Yulug

**Affiliations:** ^1^ Center for Psychiatric Neuroscience Feinstein Institute for Medical Research Manhasset New York USA; ^2^ Functional Imaging and Cognitive‐Affective Neuroscience Lab (fINCAN) Health Sciences and Technology Research Institute (SABITA), Istanbul Medipol University Istanbul Turkey; ^3^ Department of Neurology, School of Medicine Istanbul Medipol University Istanbul Turkey; ^4^ Department of Biophysics, School of Medicine Istanbul Medipol University Istanbul Turkey; ^5^ Program of Electroneurophysiology, Vocational School Istanbul Medipol University Istanbul Turkey; ^6^ Department of Neurology and Clinical Neuroscience, School of Medicine Alanya Alaaddin Keykubat University Alanya Turkey

**Keywords:** AD, rTMS, VEP, VERP

## Abstract

**Background:**

Repetitive transcranial magnetic stimulation (rTMS) has emerged as a promising alternative therapy for Alzheimer's disease (AD) due to its ability to modulate neural networks and enhance cognitive function. This treatment offers the unique advantage of enabling real‐time monitoring of immediate cognitive effects and dynamic brain changes through electroencephalography (EEG).

**Objective:**

This study focused on exploring the effects of left parietal rTMS stimulation on visual‐evoked potentials (VEP) and visual event‐related potentials (VERP) in AD patients.

**Methods:**

Sixteen AD patients were recruited for this longitudinal study. EEG data were collected within a Faraday cage both pre‐ and post‐rTMS to evaluate its impact on potentials.

**Results:**

Significant alterations were found in both VEP and VERP oscillations. Specifically, delta power in VEP decreased, while theta power in VERP increased post‐rTMS, indicating a modulation of brain activities.

**Discussion:**

These findings confirm the positive modulatory impact of rTMS on brain activities in AD, evidenced by improved cognitive scores. They align with previous studies highlighting the potential of rTMS in managing hyperexcitability and oscillatory disturbances in the AD cortex.

**Conclusion:**

Cognitive improvements post‐rTMS endorse its potential as a promising neuromodulatory treatment for cognitive enhancement in AD, thereby providing critical insights into the neurophysiological anomalies in AD and possible therapeutic avenues.

## Graphical abstract

Administering rTMS along 2 weeks to the left lateral parietal cortex of AD patients results in a significant decrease in VEP delta power, alongside a marked increase in VERP theta power. This compelling evidence broadens our understanding of rTMS's impact, pointing to it as a potentially efficacious intervention for AD patients. (The figure adapted from biorender.com).

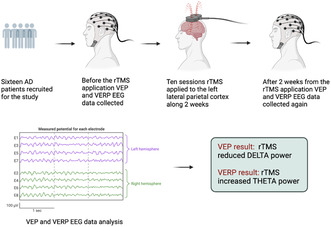

## 1 INTRODUCTION

Alzheimer's disease (AD) is the most prevalent and devastating neurodegenerative disorder leading to dementia. Although several methods have been proposed for identifying biomarkers for the early diagnosis and assessment of treatment responses in AD, including cerebrospinal fluid markers, genetic markers, and structural, functional, and metabolic metrics, their specificity for AD remains relatively limited.^1^ This suggests a need for a second set of biomarkers of disease progression and treatment response. These would directly collect the neuro‐electric activity in the brain with high temporal resolution, also enabling immediate monitoring of the therapeutic response. Several studies have shown that due to its cost‐effectiveness, easy accessibility, and lack of invasiveness, encephalography (EEG) is a particularly widely employed modality in this context.^2^, ^3^ Event‐related potentials (ERPs) permit the measurement of brain responses to specific cognitive functions^4^ and the detection of slight alterations in cognitive functions between groups. Briefly, each ERP has a specific component amplitude that relates to the intensity of the cognitive process, and a latency that relates to the time required for processing the task concerned.^4^ In this context, ERPs can be triggered during both passive and active tasks requiring either active attention to the tasks or lack thereof, respectively. There is substantial evidence that AD affects the parietal and occipital regions of the brain. An earlier analysis of the link between visual impairment and regional cerebral glucose metabolism in AD demonstrated significant hypometabolism in both the primary and secondary visual regions. In line with this finding, numerous functional neuroimaging investigations in patients with AD have shown a significantly greater loss of activations in parietal and occipital regions than in temporal regions.^5^, ^6^, ^7^ Moreover, a behavioral investigation of AD found that navigational impairment appears to be linked with a malfunction of parietal lobe‐related extrastriate visual cortex processing.^8^ Intriguingly, the broad therapeutic cholinergic strategy extensively used to treat AD seems to also reduce visual attention task‐dependent responses.^8^ For instance, physostigmine, a cholinesterase inhibitor, has been reported to improve activity in the right parietal and prefrontal cortices in patients with AD.^9^, ^10^ Similarly, improved event‐related oscillatory responses have been shown in AD following the administration of cholinergic medications.^11^

Several studies have shown that repetitive transcranial magnetic stimulation (rTMS) may represent a viable alternative to medication due to its modulatory effect on network properties, resulting in observable pro‐cognitive effects.^12^, ^13^ Furthermore, it appears to possess the significant advantage of permitting the simultaneous monitoring of the immediate effects of rTMS on cognition and dynamic brain changes through EEG, which is greatly superior in terms of temporal resolution to other dynamic imaging methods. Herein, given that the primary drawbacks of EEG recording and fMRI are inadequate spatial and temporal resolution, respectively, the combination of these two techniques has the potential to provide a holistic understanding of brain activity that surpasses the inherent limitations of each modality when used separately.^14^ To be more specific, the potential pro‐cognitive effects of rTMS might be attributed to the diverse capabilities of the dorsolateral prefrontal cortex (DLPFC)^15^ and frontoparietal networks, which play a significant role in numerous cognitive processes.^16^ Therefore, the activation of these specific brain regions and networks has the potential to enhance cortical excitability and synaptic,^17^ which can ultimately improve cognitive functions.^17^

This study investigated visual sensory‐evoked oscillations of patients with AD and healthy controls by comparing VEP and visual event‐related potentials (VERP). Given the importance of the association between these potentials and the dynamism of cognitive networks, we compared baseline VEP and VERP potentials following rTMS applications in patients with AD and evaluated the therapeutic role of rTMS on cognitive scores and specific oscillatory brain activity in such patients. Although several VEP and VERP studies have shown considerable alterations in delta and theta responses in patients with AD,^18^, ^19^, ^20^ a finding partly aligning with our own data, to the best of our knowledge no studies have evaluated the VEP and VERP correlates of delta and theta responses in patients with AD before and after rTMS application. In order to fill this gap, we set out to determine whether the pro‐cognitive effects of rTMS are associated with significantly altered theta and delta wave activities in key cognitive brain networks.

## 2 MATERIALS AND METHODS

### 2.1 Subjects

This retrospective study utilized the VEP and VERP data along with MMSE scores of 16 patients, which included 12 women. This distribution is consistent with our preceding study as delineated by Velioglu et al.^21^ The diagnosis of AD was established based on the criteria set forth by the National Institute on Aging‐Alzheimer's Association Workgroups' diagnostic guidelines for Alzheimer's disease.^22^ The Clinical Dementia Ratio (CDR) scores of the patients ranged between 1 and 2. Patients who did not provide consent, those with a history of alcohol/substance addiction, prior traumatic brain injury, or those who had experienced a serious stroke or had other neurological diseases with lasting sequelae were not included in the study.

A power analysis (G*power, ver. 3.1.6.6) established that a sample size of at least 13 patients was necessary to ensure a power of 90% and a significance level of *α* = 0.05, thus providing us with a robust statistical base to infer meaningful conclusions from our findings. The study's protocol received approval from the local Ethical Committee of Istanbul Medipol University, as documented in ethical report no. E‐10840098‐772.02‐6177. This underlines our unwavering commitment to preserving high ethical standards and guaranteeing the safety and welfare of all research participants throughout the duration of the study.

### 2.2 Neuropsychological evaluations

In our comprehensive neuropsychological assessment, we evaluated various cognitive domains. Global cognitive function was assessed using the Mini‐Mental State Examination (MMSE).^23^ Attention was gauged through the Digit Span Test.^24^ Memory assessments encompassed the Wechsler Memory Scale (WMS) and specific tests including the WMS Logical Memory (Immediate and Delayed), WMS Visual Reproduction Test (Immediate, Delayed, and Recognition),^25^ and the Oktem Verbal Memory Process Test.^26^ Language skills were evaluated using the Boston Naming Test^27, while visual and perceptual functions were determined through the Judgment of Line Orientation Test^27^ and the Benton Facial Recognition Test.^28^ Finally, executive functions were gauged using tools like abstract thinking, Semantic Fluency, the Stroop Test,^29^ and the Clock Drawing Test.^30^

### 2.3 Experimental design and TMS parameters

The study protocol was structured to span a period of 4 weeks. Prior to the application of rTMS, EEG data were collected from each patient. Following 2 weeks of TMS application, a second EEG collection was carried out for each participant. The rTMS treatment targeted the left lateral parietal cortex, the precise location being determined using seed‐based correlation functional magnetic resonance imaging (fMRI) analysis. Based on the results of seed‐based correlation analysis, the stimulation area was consistently identified as the left lateral cortex in all patients. This method allowed us to identify the most suitable stimulation site for each patient, thereby enhancing the efficacy of the treatment. TMS was applied over a course of 10 sessions, each consisting of 1640 pulses, as detailed in our previous study.^21^ This rigorous and standardized approach was essential in ensuring that each patient received consistent and comparable treatment throughout the study period.

### 2.4 EEG data acquisition

EEG recordings were collected in a dimly lit, sound‐proofed, and electrically protected Faraday cage by means of a BrainAmp enhancer DC framework. Based on the international 10–20 system, two connected ear references (A1 and A2) and 30 scalp electrodes (FP1, FP2, F7, F3, Fz, F4, F8, FT7, FC3, FCz, FC4, FT8, T7, C3, Cz, C4, T8, TP7, CP3, CPz, CP4, TP8, P7, P3, Pz, P4, P8, O1, Oz, and O2) were used for each participant according to his/her head size. EEG recordings were made with a low cut‐off of 0.01 and a high cut‐off of 250 Hz, with test rates of 500 Hz. All electrode impedances were less than 15 kΩ and ground impedances less than 1 kΩ. One reference electrode was bonded with a ratch to the left earlobe, and the other reference electrode was also attached to the front of the right earlobe. Electrooculogram (EOG) was recorded through electrodes placed on the right side of the forehead and below the left eye to identify eye movement in the EEG data.

### 2.5 EEG analysis

All EEG data were segregated into two distinctive categories: VEP and VERP records. During the pre‐processing stage, an infinite impulse response (IIR) digital filter was applied within the range of 0.01–60 Hz to eliminate both low and high frequencies. To identify eye movements, independent component analysis (ICA) was employed on the entire dataset. Subjectively, one or two components were removed from the data, considering the signal topography and horizontal‐ventral oscillations of the waves. Consequently, data were cleansed from eye movement components. Following this, artifact rejection was implemented on the segmented data. Each epoch was carefully inspected for any possible artifacts, such as muscle activity. Any epochs containing such artifacts were promptly removed. EEG analyses were executed separately for the VEP, target VERP, and non‐target VERP. In order to minimize subjectivity and maintain consistency in the data interpretation, all analyses were performed by the same individual.

Event‐related oscillation analyses were calculated for F3, F4, C3, C4, T7, T8, TP7, TP8, P3, P4, P7, P8, O1, and O2 electrodes on power‐spectrum by Brain Vision Analyzer 2.2 using wavelet transform, a formula implemented in the software. Parameters used on wavelet transform can be seen in Table 1. For delta band activity signals were analyzed on 0.5–3.5 Hz frequency in 0–600 ms and 600–1000 ms and theta band activity on 4–7 Hz frequency in 0–400 ms and 400–800 ms after the stimulation according to the grand average of all subjects. 200‐ms‐long baseline correction was applied for both frequency bands (Table 1).

**TABLE 1:** Wavelet parameters.

Wavelet function	Frequency steps	Frequency layer steps	Wavelet normalization	Morlet parameter	Normalization	Output values
Morlet Complex	30	Logarithmic	Instantaneous Amplitude (Gabor Normalization)	3	Decibel	Spectral Power ‐ Real Values [μV^2^]

### 2.6 Statistical analysis

Statistical analyses were run with Jamovi (version 2.3.21) and IBM SPSS (version 25.0) Statistics software. For the normality test, Shapiro–Wilk test was applied to neuropsychological data. The distribution didn't fit with normal distribution therefore Wilcoxon signed‐rank test was used for statistical analyses on neuropsychological data within‐subject to see the effect of TMS.

EEG data were analyzed with repeated measures ANOVA. Separate statistical analyses were run for two conditions (pre–post‐TMS) at different time intervals. 0–600 ms and 600–1000 ms time windows for delta; 0–400 and 400–800 ms time windows for theta, on 14 EEG pairs at 7 locations ([frontal (F3–F4), central (C3–C4), temporal (T7–T8), temporoparietal (TP7–TP8), parietal (P3–P4), lateral parietal (P7–P8), occipital (O1–O2)]) investigated within‐subjects. *P* value is determined as 0.05 and Sphericity Assumed corrected *p* values were reported.

## 3 RESULTS

Following the rTMS applications, neuropsychological assessment revealed an improvement in clock drawing scores (pre‐rTMS: 1.76 ± 1.68; post‐rTMS: 2.35 ± 1.5, *p* = 0.031) and visual recognition memory scores (pre‐rTMS: 1.28 ± 1.23; post‐rTMS: 1.94 ± 1.55, *p* = 0.048).

**FIGURE 1:**
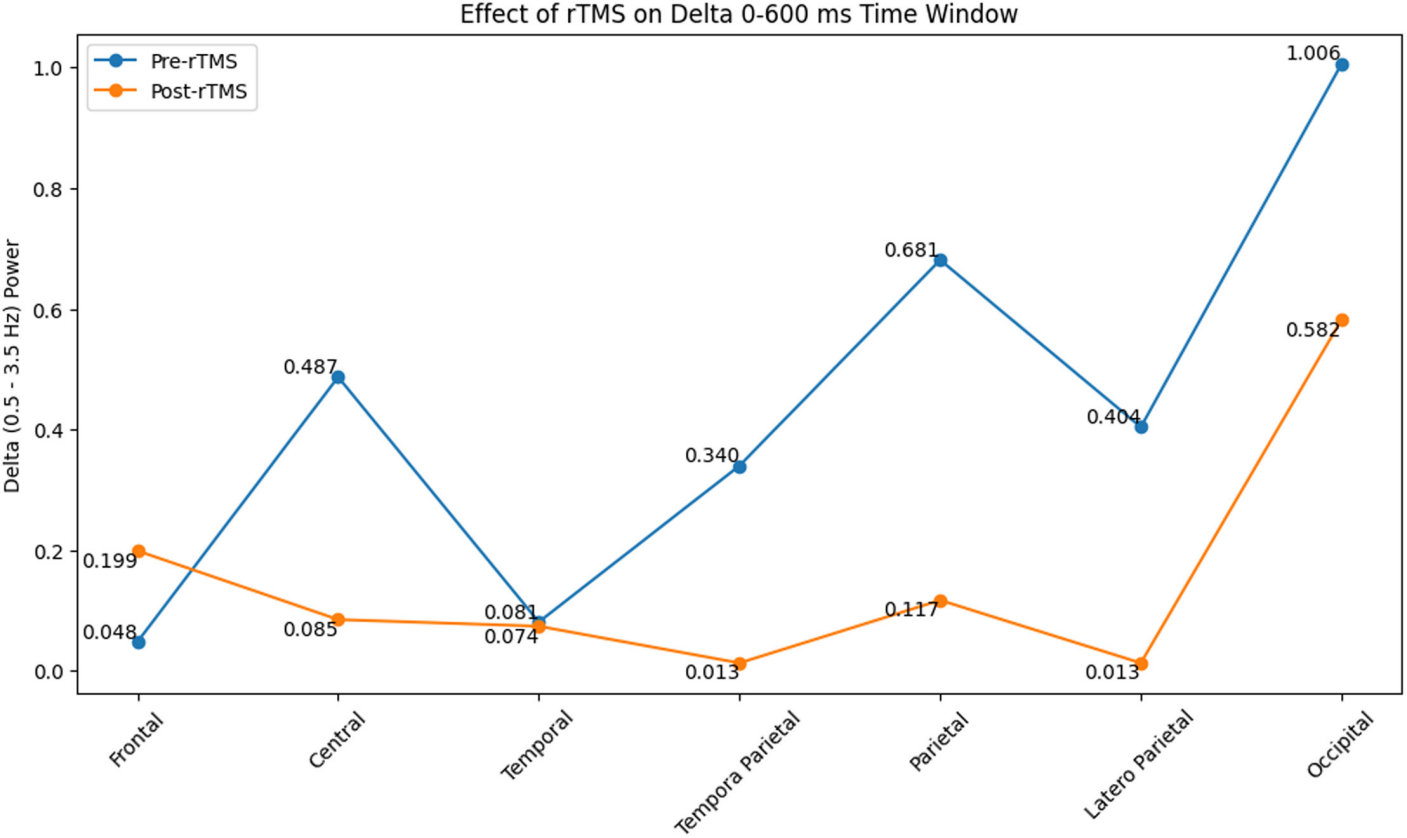
The effect of rTMS on VEP delta activity in 0–600 ms in the brain regions.

**FIGURE 2:**
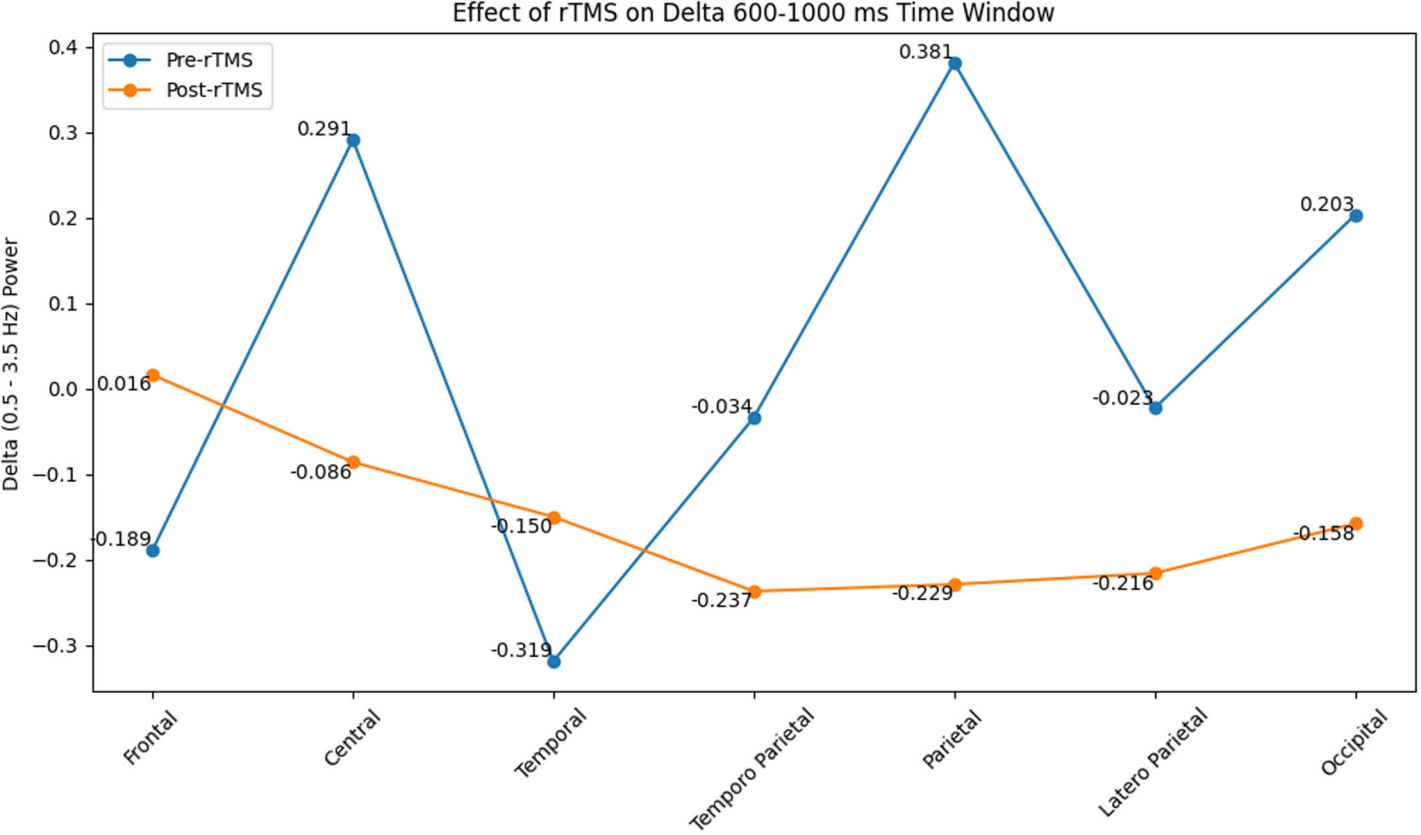
The effect of rTMS on VEP delta activity in 600–1000 ms in the brain regions.

**FIGURE 3:**
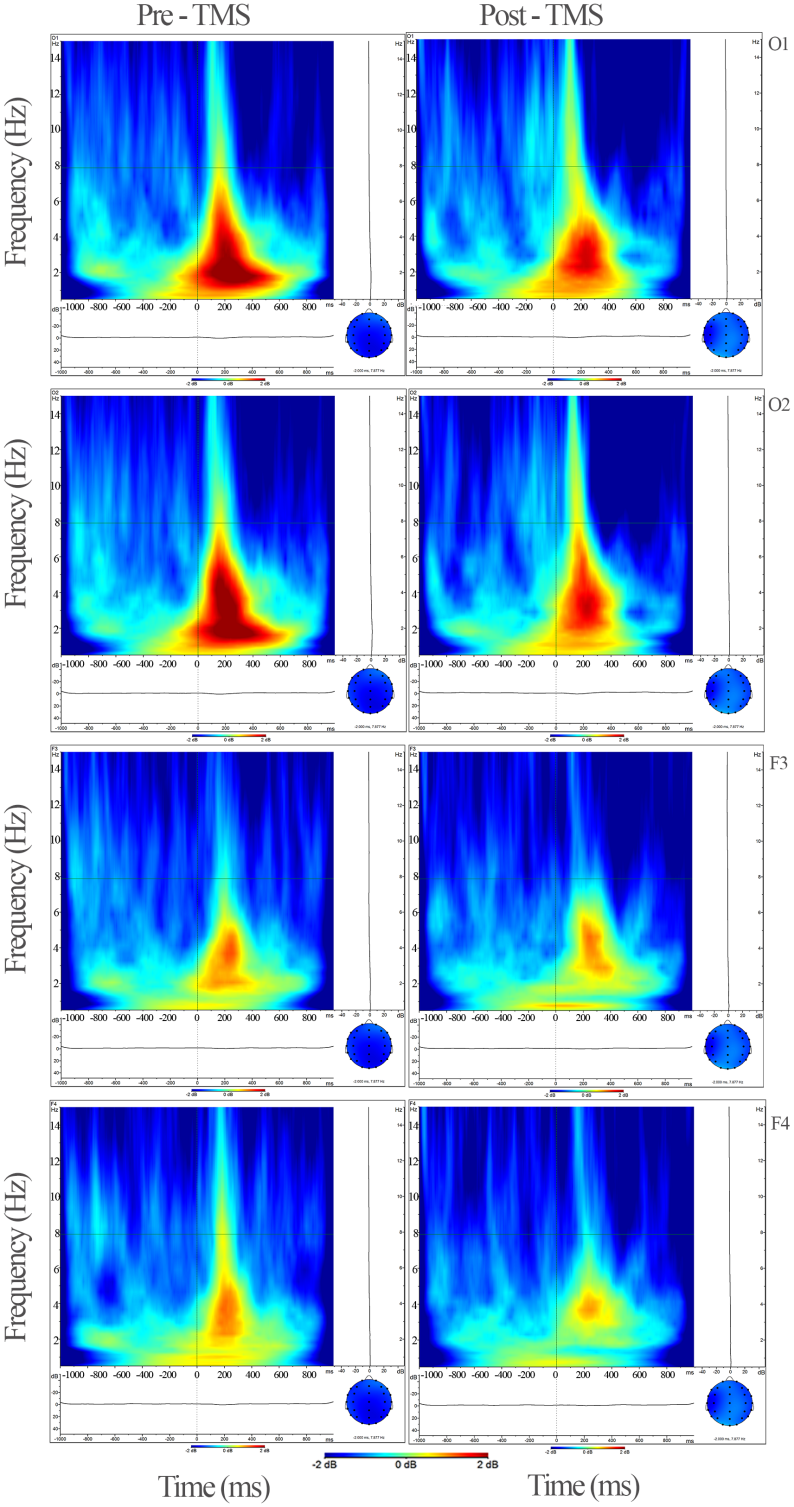
The grand average figures of evoked potential analysis for delta (0.5–3.5 Hz) in the time–frequency domain in activation to visual flash stimulation. Occipital and frontal areas are presented for both conditions (pre‐ and post‐rTMS) in the figure. Delta power decreased after rTMS on early and late time windows (*F* = 4.942, *p* = 0.042, and ηp2 = 0.248). The X‐axis represents time (−1000 ms to 1000 ms), and the Y‐axis represents frequency (0.5–15 Hz); the point at which the stimulus arrives is marked as a zero point on the X‐axis. TMS, transcranial magnetic stimulation; O1–O2, Occipital electrodes; F3–F4, Frontal electrodes.

Statistical analysis of the EEG data revealed a significant decrease in delta oscillations for different conditions of VEP oscillations from rTMS to post‐rTMS application. Specifically, a significant decrease was observed within the time range of 0–600 ms (*F* = 4.942, *p* = 0.042, and ηp2 = 0.248) (Figure 1). This reduction in delta band activity was predominantly observed in the central, temporoparietal, parietal, and occipital regions except for the temporal region during both the 0–600 ms (*F* = 3.582, *p* = 0.014, and ηp2 = 0.193) and 600–1000 ms (*F* = 3.473, *p* = 0.033, and ηp2 = 0.188) time intervals (Figures 1, 2, 3).

**FIGURE 4:**
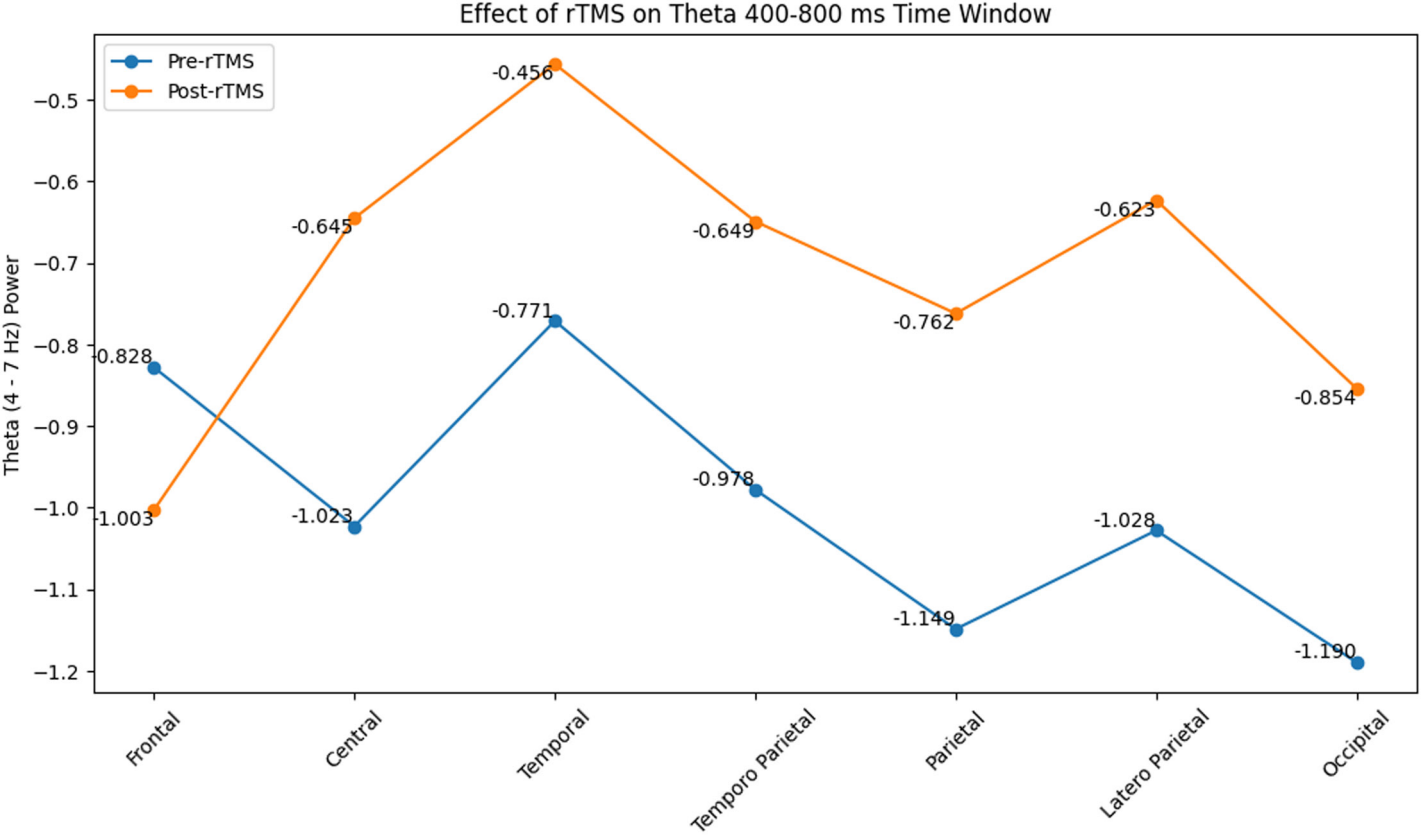
The effect of rTMS on VERP theta activity in 400–8000 ms in the brain regions.

**FIGURE 5:**
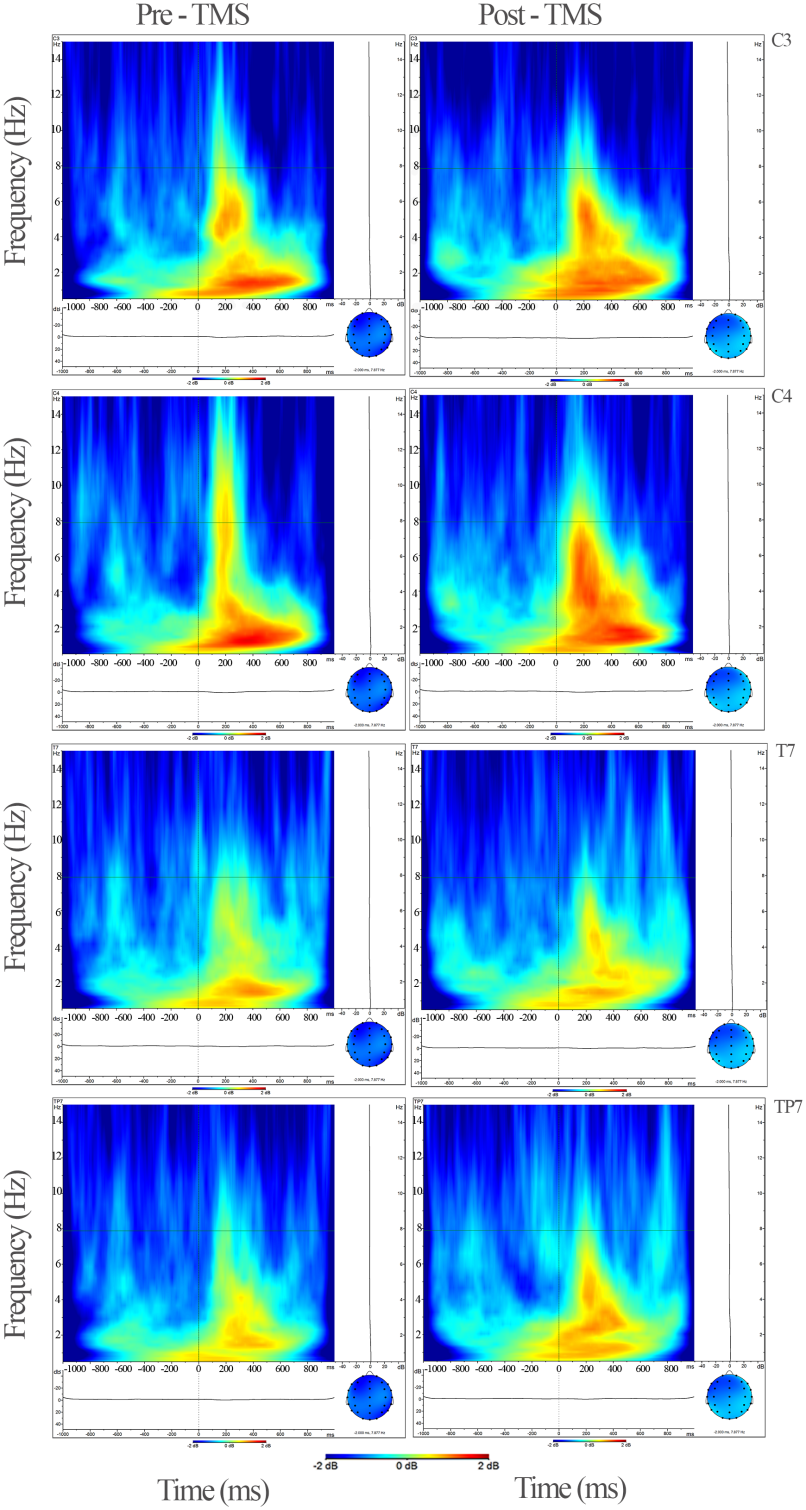
The grand average figures of event‐related analysis for theta (4–7 Hz) in time–frequency domain response in oddball paradigm non‐target stimulation. Occipital and frontal areas are presented for both conditions (pre‐ and post‐rTMS) in the figure. Theta power decreased after rTMS for all locations except the frontal region (*F* = 8.288, *p* = 0.012, and ηp2 = 0.372). The X‐axis represents time (−1000 ms–1000 ms), and the Y‐axis represents frequency (0.5–5 Hz); the point at which the stimulus arrives is marked as a zero point on the X‐axis. TMS, transcranial magnetic stimulation; C3–C4, central electrodes; T7, temporal electrodes; TP7, temporoparietal electrodes.

In the oddball paradigm, following the application of rTMS compared to baseline, an increase occurred in VERP theta activity for non‐target stimuli (*F* = 8.288, *p* = 0.012, and ηp2 = 0.372) (Figure 2). Notably, these differences were observed in all brain regions, except the frontal area (*F* = 1.288, *p* = 0.033, and ηp2 = 0.188) (Figures 4, 5).

## 4 DISCUSSION

Although sensory processing has been explored in numerous studies using various methodologies, including auditory and visual‐evoked potentials in individuals with AD,^31^, ^32^, ^33^ investigations into oscillations are relatively rare. The results of the present study showed that delta waves were significantly decreased on VEP, and that theta waves were significantly increased on VERP following rTMS application, a finding correlated with improved cognitive scores. This is also in line with a previous study indicating that VERP responses are primarily linked to cognitive functioning, while VEP responses are associated with sensory processing. Our results thus provide valuable evidence for a dissociation pattern between the circuits related to sensory and cognitive theta responses in patients with AD.^34^ Our findings also contribute to the growing body of evidence suggesting that diminished visual memory performance may be an early indicator of AD, manifesting years prior to formal diagnosis.^35^

Altered theta and delta responses have been also confirmed in several AD and Parkinson's disease studies.^36^, ^37^, ^38^ Yener et al. (2009) showed that without cognitive load, sensory visual responses for delta frequencies cannot be differentiated between AD and healthy groups, while theta band power increased in occipital regions for AD.^11^, ^33^ In another study, delta responses to visual stimuli were found to be significantly lower in Parkinson's dementia patients compared to a healthy group, which the authors explained in terms of a possible loss of visual–spatial function of early sensory processing.^39^ In contrast, an increase in the amplitude of the evoked responses on delta and theta was observed in AD in another study.^40^ In that novel study, Guidi et al. explained the increased amplitude values of theta and delta as a reflection of a hyperexcitability status of the cortex in specific areas secondary to visual processing, which may be due to a reduction in inhibitory circuits as a result of a degenerative process evident in dementia patients. To sum up, these findings align well with that EEG theta changes are an early marker of cognitive decline in neurodegenerative diseases, including especially the AD.^41^, ^42^ This fits well with a recent novel study by Wu et al. demonstrating that intermittent theta burst stimulation can alleviate symptoms and cognition in Alzheimer's disease.^17^

VERP studies have also provided strong evidence suggesting the role of delta and theta band alterations in AD. The delta band in AD has been reported to gradually decrease compared to Mild Cognitive Impairment (MCI) and healthy groups for both visual and auditory stimuli.^32^, ^43^, ^44^ Interestingly, most affected areas on the delta band overlapped with frontal and central locations and their related oddball paradigm including the target stimuli during the task.^45^, ^46^

rTMS also exerts considerable effects on evoked (theta and delta), resting‐state EEG (theta and delta), beta, and gamma responses comparable to the impact of anti‐Alzheimer medical treatment, as characterized by increased spontaneous delta and theta activity after medical treatment.^47^ A similar modulatory effect for rTMS has been also reported on sensory and cognitive circuits in patients with AD presenting with substantial differences in theta responses compared to healthy individuals.^33^, ^48^ Our findings of altered VEP responses after rTMS also fit well with a recent study by Khedrs et al. showing that reduced P300 latency‐delta frequency is a subcomponent of P300‐ EEG responses in AD that has been reversed with successful tDCS application.^49^ From that perspective, our electrophysiological data are unique in supporting previous data regarding altered delta responses in patients with AD and are consistent with the findings of Vecchio et al. reporting altered theta bands values in patients with AD that responded well to neuromodulation (tACS, tDCS) treatment.^50^ Similar results for EEG theta waves have been observed in larger samples, providing further support for a central role of theta alterations in the pathophysiology of AD, alterations which were similarly normalized with rTMS.^51^, ^52^

In addition, our findings of improved cognitive scores are worth discussing based on recent promising results concerning the effects of rTMS and tDCS on cognition. More specifically, as well as their electrophysiological effects described above, rtMS/tDCS also exhibit considerable pro‐cognitive effects, which are associated with relevant electrophysiological changes in patients with AD. For instance, high‐frequency rTMS has been shown to enhance cognition^53^ and correlated EEG activity.^54^ Our previous AD study also showed that elevated delta and theta activity on resting‐state EEG decreased after TMS.^55^ Within that context, multiple studies have provided evidence that rTMS can alter cortical excitability and may have the potential to be used as a beneficial intervention for enhancing cognitive function in various conditions, such as AD, moderate cognitive MCI, Traumatic Brain Injury (TBI), and depression.^56^, ^57^, ^58^, ^59^ Herein, through the modification of stimulation frequency, repeated transcranial magnetic stimulation rTMS possesses the capability to regulate the balance between cortical excitation and inhibition in a bidirectional manner, hence improving cognitive functions. This is in line with the impaired balance between excitation and inhibition in the pathogenesis of AD.^60^, ^61^

On the other hand, Koch et al. showed that rTMS application to the precuneus for 2 weeks primarily altered beta responses in AD.^62^ Other therapeutic data have also confirmed that rTMS at different frequencies improves cognition associated with significant alterations in VEP theta and delta potentials. Later studies, including that by Koch et al., additionally showed that single‐pulse TMS applied on the precuneus enhanced local gamma oscillations in the frontal area in patients with AD compared to sham stimulation.^63^, ^64^ These findings were confirmed by Cui et al., showing increased ERPs in the left parietal region after rTMS while subjects were solving memory test paradigms, which is consistent with our present result for VERP theta activity during the oddball paradigm.^65^ It is also worth noting that our observations of increased post‐rTMS theta responses are also partly consistent with a previous baseline study showing decreased theta power in patients with AD.^1^, ^11^, ^33^

Overall, our findings are in line with those of several studies evaluating the effects of tDCS and anti‐dementia medical treatments at EEG. More specifically, our findings of improved clock drawing and visual recognition memory scores in particular align with our recent research^21^ described above, and accord with most studies in this field that evaluated the efficiency of TMS & tDCS through resting‐state EEG, cognitive scores, and metabolic changes in the treatment of AD.^66^, ^67^ A good example of this is a recent study showing that rTMS over the left and right DLPFC either improved or stabilized cognitive function in patients with MCI and early dementia.^58^ These results were also confirmed by Zhang et al., who identified the left DLPFC as the optimal location for rTMS application, based on the most effective cortical metabolic changes seen on the left DLPFC after rTMS.^68^ Our finding of a significant association between high theta band activity and good working memory scores is in line with previous tDCS studies showing that increased ERP in the theta and alpha range is associated with improved N‐Back scores.^69^, ^70^ This also accords well with previous research showing increased theta power and P200 amplitude^71^ associated with improved cognitive scores after tACS and tDCS applications on right and left DLPFC.^72^, ^73^

From a baseline pathophysiological perspective, our findings of altered VEP delta and VERP theta activities may also suggest that sensorial and cognitive processes are distinct from one another in terms of the perception and interpretation or recognition of visual cognitive processes.^11^, ^33^, ^74^, ^75^ This novel hypothesis has been suggested in several studies showing a significant association between P100, N100 and P200, P300, and delta and the early stage of sensory processing compared to the cognitive component of visual stimuli reflected in late P300, N400, theta, and gamma frequency bands.^18^, ^76^, ^77^

Although the study findings presented herein may offer significant contributions to translational Alzheimer's investigations, limitation of this study includes its small sample size and retrospective design.

## 5 CONCLUSION

To summarize, our findings support the idea that EEG event‐related theta responses may respond well to neuromodulation therapies (Cespón et al., 2019; Güntekin et al., 2020). However, the altered EEG dynamics in AD seem not to be limited to theta and delta bands. The localization of rTMS‐oriented treatments may be critical in eliciting valuable responses at different frequencies that might result in a substantial pro‐cognitive effect. Although our findings provide valuable evidence for the therapeutic role of rTMS in cognition and VEP dynamics, further research involving more patients and larger sample groups is now needed. This is especially true when considering the divergent findings reported in the literature, which may be attributable to methodological differences, including the different rTMS frequencies selected for the therapeutic framework.

## AUTHOR CONTRIBUTIONS

HAV collected the complete EEG data from patients, coordinated the analysis, and contributed to the drafting and review of the manuscript. ZEZD analyzed the EEG data, interpreted the results, and participated in the drafting of the manuscript. LH was involved in patient recruitment for the study and provided valuable input during manuscript review. BG and TA supervised the EEG data collection and contributed to the EEG analysis. Meanwhile, BY conducted the statistical analysis of the results and took the lead in both drafting and reviewing the manuscript.

## CONFLICT OF INTEREST STATEMENT

All authors report no conflict of interests. We confirm that we have read the Journal's position on issues involved in ethical publication and affirm that this report is consistent with these guidelines.

## Data Availability

The data that support the findings of this study are available from the corresponding author upon reasonable request.
